# GSK3β-dependent lysosome biogenesis: An effective pathway to mitigate renal fibrosis with LM49

**DOI:** 10.3389/fphar.2022.925489

**Published:** 2022-09-26

**Authors:** Jinhong Ren, Huizhi Wei, Jian Sun, Xiue Feng, Yuanlin Zhang, Hongxia Yuan, Junqiu Miao, Xiaoming Qi, Yuanbiao Qiao, Baoguo Xiao, Qingshan Li

**Affiliations:** ^1^ School of Pharmaceutical Science, Shanxi Medical University, Taiyuan, China; ^2^ Shanxi Key Laboratory of Innovative Drug for the Treatment of Serious Diseases Basing on the Chronic Inflammation, College of Traditional Chinese Medicine and Food Engineering, Shanxi University of Chinese Medicine, Taiyuan, China

**Keywords:** LM49, renal fibrosis, extracellular matrix, lysosome biogenesis, glycogen synthase kinase 3β

## Abstract

Renal fibrosis is an incurable disorder characterised by an imbalance of the extracellular matrix (ECM) favouring excess production over degradation. The identification of actionable pathways and agents that promote ECM degradation to restore ECM homeostasis may help mitigate renal fibrosis. In this study, we identified 5,2′-dibromo-2,4′,5′-trihydroxydiphenylmethanone (LM49), a compound we previously synthesised, as a small-molecule inducer of ECM degradation. LM49 administration efficiently reduced ECM deposition in renal tissue of diabetic nephropathy rats and in transforming growth factor β-treated renal fibroblast cells. LM49 promoted the cytosol-to-nucleus translocation of transcription factor EB (TFEB) to increase lysosome biogenesis, leading to lysosome-based degradation of the ECM. TFEB-mediated lysosome biogenesis was induced by LM49 directly inhibiting the activity of glycogen synthase kinase 3β (GSK3β) rather than mammalian target of rapamycin complex 1. LM49 inhibited GSK3β kinase activity concentration-dependently via competing with ATP. Direct binding between LM49 and GSK3β was confirmed by the bio-layer interferometry assay, cellular thermal shift assay, and drug affinity responsive target stability. A molecular docking and molecular dynamic simulation revealed that LM49 occupied the ATP pocket of GSK3β, which was consistent with the kinase activity assay. In summary, LM49 enhances TFEB-mediated lysosome biogenesis by directly inhibiting GSK3β, leading to the degradation of the ECM by lysosomes. The enhancement of GSK3β-dependent lysosome biogenesis to rebalance the ECM may be a novel strategy to counteract renal fibrosis, and LM49 may be a viable clinical candidate for treating this disorder.

## Introduction

Renal fibrosis occurs during several types of chronic kidney disease (CKD), including diabetic nephropathy (DN), leading to progressive and irreversible loss of renal function in a process known as end-stage renal disease. Renal fibrosis is principally characterised by the presence and proliferation of myofibroblasts and an excessive accumulation of extracellular matrix (ECM) proteins (Muñoz-Félix et al., 2015). Myofibroblasts originate from several types of cells. However, although the relative contribution of cell origin is still controversial, resident fibroblasts are an important sources of myofibroblasts (Kuppe and Ibrahim, 2021).

Transforming growth factor-β1 (TGF-β1), a master regulator of renal fibrosis, induces terminal differentiation of fibroblasts into myofibroblasts, which secrete additional ECM components (such as collagen one and fibronectin) (Gu et al., 2020). TGF-β1 induces excess ECM production over degradation, disrupting the ECM homeostasis. Anti-TGF-β1 drugs can reduce TGF-β1-induced ECM production that inhibit TGF-β1 synthesis and activation, binding of active TGF-β1 to TGF-β1 receptor (Tβ1R), Tβ1R function, or signalling downstream of Tβ1R (Zhao et al., 2019). To date, however, direct targeting of TGF-β1 has led to more toxicity than benefit due to the involvement of TGF-β1 in other biological processed, such as inflammation and immune (Meng et al., 2016). Promoting ECM degradation has been investigated as a pharmacologically actionable pathway to mitigate fibrosis (Zhao et al., 2019). Indeed, peroxisome proliferator-activated receptor (PPAR) agonists fenofibrate (Kang et al., 2015) and troglitazone (Routh et al., 2002; Weigert et al., 2003), have been approved by FDA to mitigate renal fibrosis, as PPAR signalling promotes fibroblast-mediated ECM degradation (Zhao et al., 2019).

ECM degradation is a sequential process comprising extracellular protease-mediated cleavage, intracellular uptake, and lysosomal degradation of the components (Zhao et al., 2019). Indeed, lysosomes have a pivotal role in maintaining homeostasis at the cellular and organismal level (Ballabio and Bonifacino, 2020). Meanwhile, enhanced lysosome biogenesis is an endolysosomal damage-response mechanism, used by cells to cope with lysosomal dysfunction (Meyer-Schwesinger, 2021). Thus, targeting lysosomal biogenesis is an effective approach to modulate lysosomal degradation and enhance cellular clearance, in order to ameliorate lysosomal storage diseases (LSDs) (Sardiello et al., 2009). However, the role of lysosome biogenesis in ECM degradation is still unknown. Nearly all genes involved in lysosome biogenesis are under the transcriptional control of transcription factor EB (TFEB), which in turn is negatively regulated by the mechanistic target of rapamycin complex 1 (mTORC1) (Bajaj et al., 2019). Phosphorylation of TFEB by mTORC1 inhibits TFEB shuttling into the nucleus. When mTORC1 is inhibited, TFEB is dephosphorylated and translocates into the nucleus, thereby triggering the expression of lysosomal genes (Zhang et al., 2020). Independently of mTORC1, mitogen-activated protein kinase 1 (MAPK1 or ERK) (Settembre et al., 2011), protein kinase (PKB or Akt) (Palmieri et al., 2017), and glycogen synthase kinase (GSK3β) (Li et al., 2016) have also been reported to control TFEB activity and localisation. The modulation of lysosome biogenesis, influencing lysosome-based degradation of ECM, may be an alternative route to ameliorate renal fibrosis.

5,2′-dibromo-2,4′,5′-trihydroxydiphenylmethanone (LM49) is a polyphenol derivative synthesised by our group from marine plants. Curcumin, resveratrol and other polyphenol compounds have shown renal protective roles, through prevention of inflammatory molecule release and reduction of the deposition of ECM at the priming and activation stage of renal fibrosis; this has been shown both *in vitro* and *in vivo* (Sun et al., 2017; Den Hartogh and Tsiani, 2019; Zhang et al., 2019). LM49 exerted anti-inflammatory (Yang et al., 2019; Yuan et al., 2019) and immunomodulatory activity (Yang et al., 2020), and also showed therapeutic effects in acute pyelonephritis (Zhang et al., 2018), which suggest that LM49 may be a potential therapeutic candidate for the treatment of renal fibrosis. In this study, the therapeutic effect, the mechanism and the potential target of LM49 on renal fibrosis were explored.

## Materials and methods

### Antibodies and reagents

LM49 was synthesised as previously described (Zhao et al., 2010). Recombinant TGF-β1 was purchased from R&D systems (Minneapolis, MN, United States). Cycloheximide (CHX) was purchased from Selleck (Beijing, China). Bafilomycin A1, MG132, and LiCl were purchased from Sigma (St. Louis, MO, United States). Torin1 and SB415286 were purchased from MCE (Beijing, China). Antibodies against COL1, FN, and LAMP1 were purchased from Abcam (Cambridge, MA, United States). Antibodies against pS6K, S6K, pGSK-3β (Ser9), and GSK-3β were purchased from Cell Signaling Technology (Beverly, MA, United States). Antibodies against TFEB and TFE3 were purchased from Novus (Littleton, CO, United States). Antibodies against GAPDH and H3 were purchased from Proteintech (Wuhan, China). The probe LysoTracker Green was purchased from Yeasen (Shanghai, China). The Hoechst 33,342 probe and Lipofectamine 2000 reagent were purchased from Invitrogen (Carlsbad, CA, United States). Recombinant GSK-3β was purchased from Carna (Kobe, Japan).

### Animals and treatment

The animal procedures were approved by the Animal Policy and Welfare Committee of the China Institute for Radiation Protection (CIRP-IACUC-(G)2020106). Six-week-old pathogen-free male Sprague–Dawley rats (180–220 g) were obtained from Charles River Laboratories (Beijing, China). All rats were randomly divided into three groups: control group (n = 8), streptozotocin (STZ)-induced diabetic group (Diabetic group, n = 8) and STZ-induced diabetic group treated with LM49 (LM49 group, n = 8). The diabetic rats were induced by a single intraperitoneal injection of 25 mg/kg STZ dissolved in 100 mM citrate buffer. The control group received the same volume of citrate buffer. Rats with a fasting-blood glucose >16.7 mM were considered as diabetic. The urine was collected using metabolic cages to measure volume and protein concentration weeks 2–4 after STZ treatment. A 24 h urinary protein level ≥30 mg/24 h was considered to confirm DN. After DN establishment, rats were treated with 22.5 mg/kg LM49 by oral administration once a day for 6 weeks. Rats in the control and diabetic groups were administered the same volume of phosphate buffered saline. Ten weeks after STZ treatment, rats were euthanised under anaesthesia.

### Histology and immunohistochemical analysis

Kidney tissues were fixed in 4% formalin, embedded in paraffin, and cut into 4 μm-thick sections and stained with Masson’s trichrome. Masson’s trichrome staining was used to assess the collagen deposition in the obstructed kidney tissues.

For immunohistochemical staining, briefly, the slides were blocked with 5% BSA for 1 h. After incubation with primary antibodies against COL1 and FN at 4°C overnight, the slides were probed with an HRP-labelled secondary antibody for 30 min and analysed using a DAB assay kit. Images were obtained using an ECLIPSE Ti2 microscope (Nikon, Tokyo, Japan).

### Cell culture and treatment

The renal fibroblast cell line NRK49F was purchased from Cell Culture Centre, Chinese Academy of Medical Sciences and Peking Union Medical College (Beijing, China). NRK49F cells were cultured in DMEM-Ham’s medium (Gibco, NY, United States) supplemented with 10% FBS (Gibco, NY, United States). The cells, after reaching approximately 80% confluence, were pre-treated with LM49 for 4 h, followed by incubation with recombinant TGF-β1 (5 ng/ml) for the indicated time.

### Immunoblotting

Cells were lysed in RIPA buffer (50 mM Tris-HCl, pH 7.4, 150 mM NaCl, 1% NP-40, 0.5% sodium deoxycholate) containing Complete Protease Inhibitor Cocktail and Phosphatase Inhibitor Cocktail (Roche, Basel, Switzerland). Lysates were centrifuged at 12,000 rpm for 15 min and quantified using the BCA protein assay kit (Boster, Wuhan, China). Total proteins were resolved by SDS-PAGE and subsequently transferred to polyvinylidene difluoride membrane. The membrane was then respectively incubated with specific primary antibodies and secondary antibodies. Images were detected by Amersham Imager 600 (Cytiva, DC, United States).

### Quantitative real-time PCR

Total RNA was extracted from cells using TRIzol Reagent, according to the manufacturer’s instruction (Takara, Tokyo, Japan). cDNA was synthesised with a reverse-transcription kit in a 20 μl reaction mixture (Takara, Tokyo, Japan). The levels of gene expression were quantified using SYBR Premix Ex Taq (Takara, Tokyo, Japan) and 7900HT Real-Time PCR system (Applied Biosystems, MA, United States). The mRNA levels of targeted genes were normalised with that of *Gapdh.* The primers of target genes were shown in Supplementary Table S1.

### Immunofluorescence staining

Cells were seeded in 96-well plates for each group with three wells. After incubation with LM49 or TGF-β1 for the indicated time, cells were fixed in 4% paraformaldehyde at room temperature (RT) for 10 min and permeabilised with 0.1% Triton X-100 in PBS at RT for 5 min. After blocking with 1% BSA at RT for 30 min, cells were incubated with primary antibodies in buffer (1% BSA, PBS) overnight at 4°C. Alexa Fluor 488 or 647-Secondary antibodies were subsequently incubated at RT for 1 h. Images were taken for each well with nine sites at 488 and 647 nm using ImageXpress Micro four High Content Imaging System with Ph1 S Plan Fluor ELWD ADM 20XC objective and Andor SDK3 camera (Molecular Devices, CA, United States). For quantitation of colocalization, images of a mean of twenty-seven sites for three wells per experiment were analysed using MetaXpress analysis software. Three independent experiments were performed.

### Lysotracker Green stain

Cells were seeded in 96-well plates for each group with three wells. After incubation with LM49 or TGF-β1 for the indicated time, cells were incubated with the Lysotracker Green dye at 37°C for 30 min. Then the medium was aspirated and the cells were quickly washed twice with PBS to remove the unbound dye. Images were taken for each well with nine sites at 504 nm using ImageXpress Micro four High Content Imaging System with Ph1 S Plan Fluor ELWD ADM 20XC objective and Andor SDK3 camera (Molecular Devices, CA, United States). For quantitation of intensity, images of a mean of twenty-seven sites for three wells per experiment were analysed using MetaXpress analysis software. Three independent experiments were performed.

### Small interfering RNA knockdown and transfection

TFEB siRNA (5′-GCA​GGU​UCA​ACA​UCA​AUG​ATT-3′), TFE3 siRNA (5′-GCC​UGU​GUC​AGG​AAA​UCU​ATT-3′), and GSK-3β siRNA (5′-CUG​CCA​UCG​AGA​CAU​UAA​ATT-3′) were purchased from GenePharma (Shanghai, China). Cells were transfected with 20 or 150 p.m. of targeted siRNA in 96-well or 6-well plates respectively using GP-transfect-Mate (GenePharma, Shanghai, China). The knockdown effect of siRNA was evaluated by Immunoblotting or qRT-PCR.

### Plasmids and transfection

The plasmid pcDNA 3.1-GSK3β was constructed by inserting the coding sequence of rat GSK3β into the vector of pcDNA3.1. Cells were transfected with pcDNA3.1 or pcDNA 3.1-GSK3β in 6-well plates using Lipofectamine 2000.

### *In vitro* kinase assay

Kinase activity was measured by Off-chip Mobility Shift Assay in Carna Biosciences. The 4× substrate/ATP/metal solution was prepared with kit buffer (20 mM HEPES, 0.01% Triton X-100, 5 mM DTT, pH 7.5), and 2× kinase solution was prepared with assay buffer (20 mM HEPES, 0.01% Triton X-100, 1 mM DTT, pH 7.5). The 5 μl of 4× compound solution, 5 ml of 4× substrate/ATP/metal solution, and 10 ml of 2× kinase solution were mixed and incubated in a well of a polypropylene 384 well microplate for 1 h at RT. Then, 70 ml of termination buffer (QuickScout Screening Assist MSA; Carna Biosciences) was added to the well. The reaction mixture was applied to LabChip™ system (Perkin Elmer), and the product and substrate peptide peaks were separated and quantitated. The kinase reaction was evaluated by the product ratio calculated from peak heights of product (P) and substrate (S) peptides (P/(P+S)).

### Enzyme kinetics assay

Enzyme kinetics was measured by Kinase-Glo Luminescent Kinase Assay and ADP-Glo Kinase Assay (Promega, WI, United States). The 5 μl of different concentrations of LM49 solution, 2.5 μl different concentrations of ATP solution, 2.5 μl of GSK3β solution, and 10 μl Kinase-Glo Reagent were mixed and incubated in a well of a solid white 96-well plate for 30 min at 30°C. Then, 20 μl of ADP-Glo Reagent was added to the well and incubated at room temperature for 40 min. Finally, 40 μl of Kinase Detection Reagent was added to the well and incubated at room temperature for 30 min. Luminescence was measured using a SpectraMax i3X multifunctional microplate reader (Molecular Devices, CA, United States). Lineweaver-Burker plot was generated by 1/V and 1/[ATP].

### Bio-layer interferometry assay

The biotinylated GSK-3β was immobilised onto Super Streptavidin (SSA) biosensors, and a duplicate SSA sensor incubated in the protein buffer was used as negative binding control. The assay was determined in 96-wells black plates at different concentrations of LM49 and PBS as a nonspecific interaction control. The binding event was recorded according to the shift in the interference pattern of light. Next, ForteBio Data Analysis was used to calculate the association and dissociation rates using 1:1 binding model, and K_D_ was represented by the ratio k_off_/k_on_.

### Molecular docking and molecular dynamics simulation

The crystal structure of GSK3β was obtained from the RCSB Protein Data Bank (PDB ID:6AE3 and 4NM3) (Stamos et al., 2014; Kim et al., 2018). The structure of LM49 was set up by X-ray and optimised to minimal energy. Autodock 4.2, with MGL tools 1.5.6, was applied to analyse the binding mode of LM49 and GSK3β, and residual interaction at the GSK3β/LM49 interface were evaluated using LigPlot. Molecular dynamics simulation was performed by Gromacs 5.1.5 using the Amber 99sb force field, and the binding free energy of LM49 with GSK3β was calculated by molecular mechanics Poisson–Boltzmann surface area (MM/PBSA).

### Statistics

Data were analysed with Prism (GraphPad Software) and presented as mean values ±standard error of the mean (SEM). Statistical analyses of different groups were performed using two-tailed Student’s *t*-test or one-way analysis of variance (ANOVA) followed by LSD test (equal variances assumed) or Dunnett’s T3 test (equal variances not assumed). *p* < 0.05 was considered statistically significant.

## Results

### LM49 ameliorates renal fibrosis in DN rats

**FIGURE 1:**
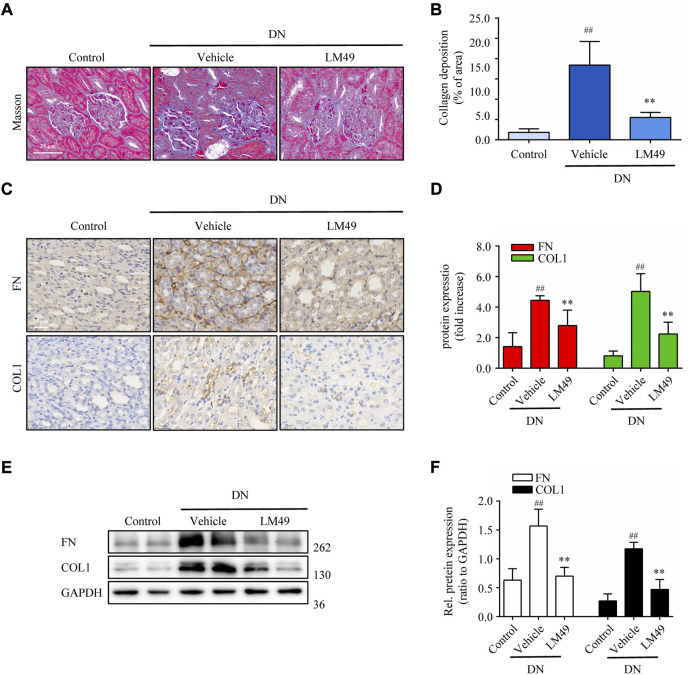
LM49 attenuates renal fibrosis in DN rats. **(A)** Representative micrographs of Masson’s trichrome staining in indicated groups. (Scale bar, 25 μm). **(B)** Quantification of collagen deposition in kidney tissue. ^##^ *p* < 0.01 compared with control group, **p < 0.01 compared with vehicle group (n = 3). **(C)** Representative micrographs of immunohistochemical staining (IHC) for FN and COL1 in indicated groups. (Scale bar, 25 μm) **(D)** Quantification of IHC staining of **(C)**, ^##^ *p* < 0.01 compared with control group, **p < 0.01 compared with vehicle group (n = 3). **(E)** Immunoblot analysis of the protein expression of FN and COL1 of kidney tissues separated from indicated groups. **(F)** Quantification of FN and COL1 intensity in **(E)**. ^##^ *p* < 0.01 compared with Control group, **p < 0.01 compared with Vehicle group (n = 3).

DN rats exhibited marked collagen deposition in renal tissue stained with Masson’s trichrome, which was significantly reduced by treatment with LM49 (Figures 1A,B). The expression of fibronectin (FN) and collagen 1 (COL1) in DN rats were remarkably increased, as visualised by immunohistochemical staining and immunoblotting assay. Similarly, LM49 significantly inhibited the upregulation of FN and COL1 protein (Figures 1C–F). Thus, LM49 ameliorates renal fibrosis in DN rats.

### LM49 reduces TGF-β1-induced ECM protein deposition *in vitro* through lysosomal protein degradation

**FIGURE 2:**
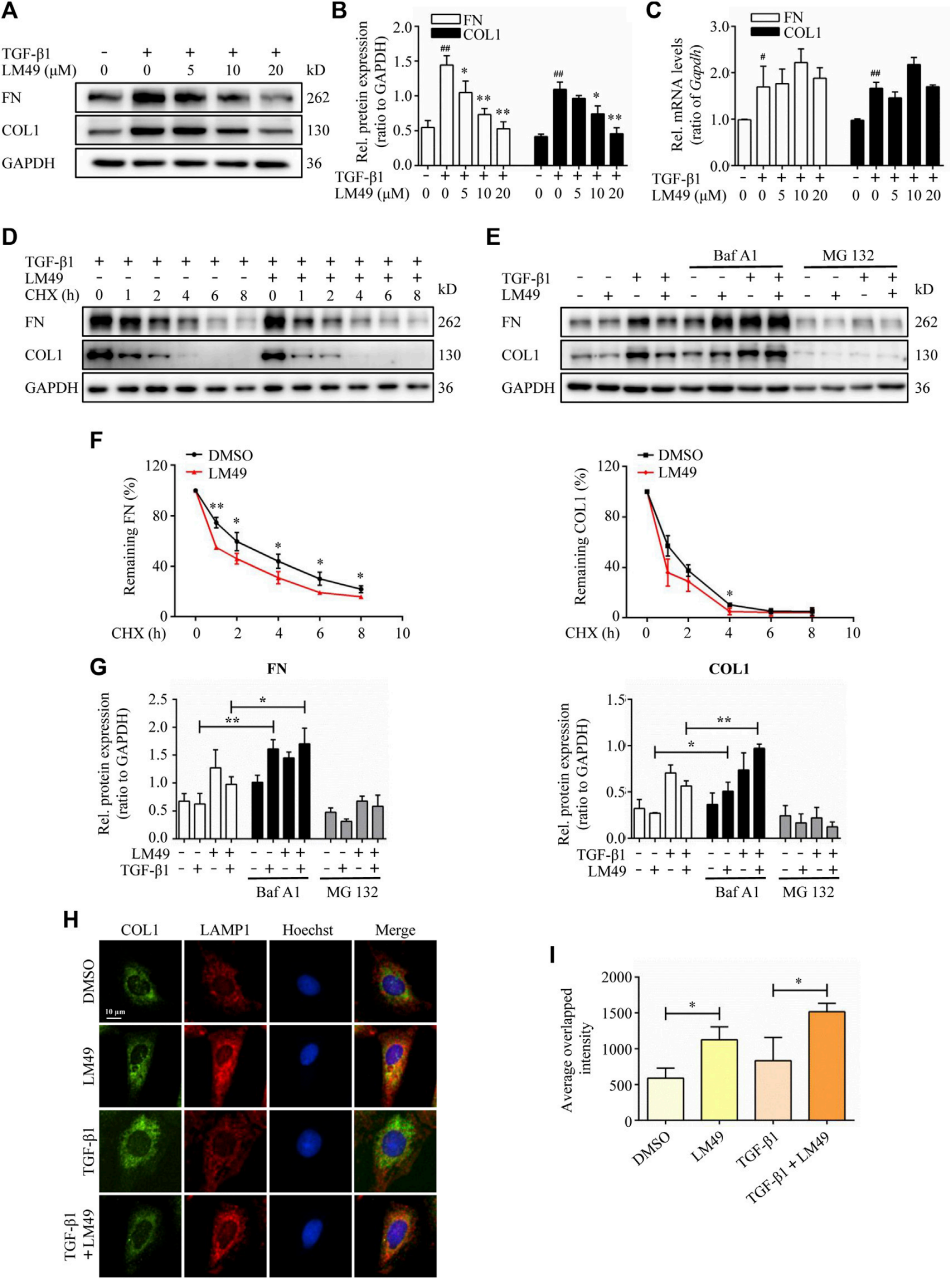
LM49 reduces TGF-β1-induced ECM protein deposition in NRK49F cells through lysosomal protein degradation pathway. **(A)** Immunoblot analysis of the protein expression of FN and COL1 in NRK49F cells treated with indicated doses of LM49 and TGF-β1 (5 ng/ml) for 24 h. **(B)** Quantification of FN and COL1 intensity in **(A)**. ^##^ *p* < 0.01 compared with DMSO group, *p < 0.05, **p < 0.01 compared with TGF-β1 group (n = 3). **(C)** Quantitative RT-PCR analysis of the mRNA levels of FN and COL1 in NRK49F cells treated with indicated doses of LM49 and TGF-β1 (5 ng/ml) for 24 h ^#^ *p* < 0.05, ^##^ *p* < 0.01 compared with DMSO group (n = 3). **(D)** Immunoblot analysis of the protein expression of FN and COL1 in NRK49F cells treated with DMSO or LM49 (20 μM) in the presence of CHX (20 μg/ml) and TGF-β1 (5 ng/ml) for the indicated time points. **(E)** Immunoblot analysis of the protein expression of FN and COL1 in NRK49F cells treated with DMSO or LM49 (20 μM) and BafA1 (10 nM) or MG132 (100 nm) in the absence or presence of TGF-β1 (5 ng/ml) for 24 h. **(F)** Quantification of FN and COL1 intensity in **(D)**. The abundance was normalised to GAPDH; each group was normalised as a percentage of that at 0 h *p < 0.05, **p < 0.01 compared with DMSO group. (n = 3). **(G)** Quantification of FN and COL1 intensity in **(E)**. *p < 0.05, **p < 0.01 compared with LM49 group or LM49+TGF-β1 group (n = 3). **(H)** Co-immunofluorescence staining for COL1 (green) and LAMP1 (red) in NRK49F cells treated with DMSO or LM49 in the absence or presence of TGF-β1 (5 ng/ml) for 24 h (Scale bar, 10 μm). **(I)** Quantification of the average overlapped intensity of **(H)**. *p < 0.05 compared with DMSO group or TGF-β1 alone treated group (n = 3).

To further explore the mechanism underlying the antifibrotic effect of LM49, we established a TGF-β1-induced fibrosis model in NRK49F cells. Immunoblot analysis revealed that LM49 decreased TGF-β1-induced FN and COL1 protein expression in a dose-dependent manner (Figures 2A,B), but not mRNA expression (Figure 2C). Therefore, LM49 may diminish TGF-β1-induced ECM protein expression at post-translational level. To directly test whether LM49 affects ECM protein stability, NRK49F cells were treated with LM49 in the presence of cycloheximide (CHX) to block new protein synthesis and ECM protein degradation was examined. After exposure to CHX, FN and COL1 became unstable and degraded rapidly in LM49-treated cells (Figures 2D–F), supporting the hypothesis that LM49 reduces ECM protein stability.

There are two major protein degradation systems: the lysosomal protein degradation system and the proteasome system. To explore which route is involved in LM49-induced ECM protein instability, we utilised bafilomycin A (BafA1), a specific inhibitor of the vacuolar type H + -ATPase within the lysosome (Mauvezin and Neufeld, 2015), and MG132, an inhibitor of the proteasome system. As shown in Figures 2E–G, the expression of FN and COL1 was significantly increased in NRK49F cells by BafA1 treatment and further increased upon stimulation of LM49 and TGF-β1. By contrast, treatment with MG132 failed to significantly increase ECM protein expression. Thus, the lysosomal protein degradation pathway, but not the proteasome system, may account for the mechanism underlying the antifibrotic effect of LM49.

We next determined whether intracellular ECM proteins were colocalised with lysosomal-associated membrane protein 1 (LAMP1), a lysosomal marker. As shown in Figures 2H,I, co-immunofluorescence staining for COL1 (green) and LAMP1 (red) demonstrated that the average overlapped intensity of green and red was significantly increased after treatment with LM49. Taken together, our data suggest that TGF-β1-induced ECM protein deposition is reduced by LM49 through the intracellular lysosomal protein degradation in NRK49F cells.

### TFEB-dependent lysosome biogenesis is required for LM49-induced ECM protein degradation

**FIGURE 3:**
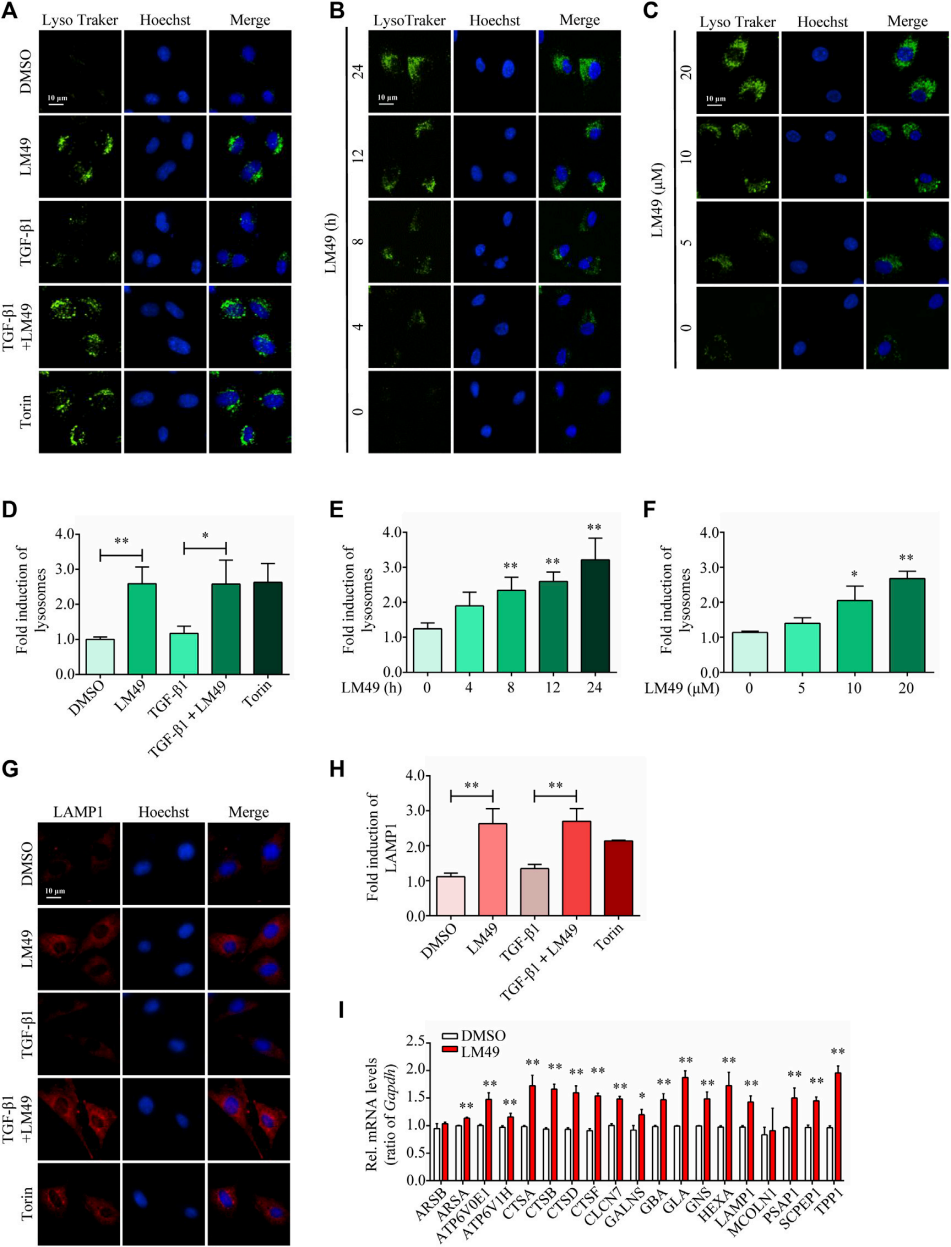
LM49 increases the lysosome biogenesis and upregulates lysosome-related genes. **(A)** LysoTracker Green staining in NRK49F cells treated with LM49 (20 μM) in the absence or presence of TGF-β1 (5 ng/ml) for 24 h or Torin1 (1 μM) for 3 h (Scale bar, 10 μm). **(B)** LysoTracker Green staining in NRK49F cells treated with LM49 (20 μM) for the indicated time points. (Scale bar, 10 μm). **(C)** LysoTracker Green staining in NRK49F cells treated with indicated doses of LM49 for 24 h (Scale bar, 10 μm). **(D)** Quantification of LysoTracker Green intensity of **(A)**. *p < 0.05, **p < 0.01 compared with DMSO group or TGF-β1 alone treated group (n = 3). **(E,F)** Quantification of LysoTracker Green intensity of **(B,C)**. *p < 0.05, **p < 0.01 compared with DMSO group (n = 3). **(G)** Immunostaining of endogenous LAMP1 in NRK49F cells treated with LM49 (20 μM) in the absence or presence of TGF-β1 (5 ng/ml) for 24 h or Torin1 (1 μM) for 3 h (Scale bar, 10 μm). **(H)** Quantification of LAMP1 intensity of **(G)**. **p < 0.01 compared with DMSO group or TGF-β1 alone treated group (n = 3). **(I)** Quantitative RT-PCR analysis of the mRNA levels of lysosome-related genes in NRK49F cells treated with DMSO or LM49 (20 μM) for 24 h *p < 0.05, **p < 0.01 compared with DMSO group (n = 3).

Lysosomes are degradation and signalling centres that coordinate cellular metabolism with clearance (Lawrence and Zoncu, 2019). Consistent with the mTOR inhibitor Torin1, LysoTracker staining intensity was increased by LM49 (Figures 3A,B), but not by TGF-β1. Moreover, LM49 induced a time-and concentration-dependent increase in LysoTracker staining (Figures 3B,C,E,F). Similarly, LM49 and Torin1 enhanced the number of lysosomes for LAMP1, but TGF-β1 did not have this effect (Figures 3G,H). In agreement with the staining results, LM49 upregulated lysosome-related genes, including *lamp 1* (Figure 3I). Thus, LM49 induces biogenesis of functionally normal lysosomes.

**FIGURE 4:**
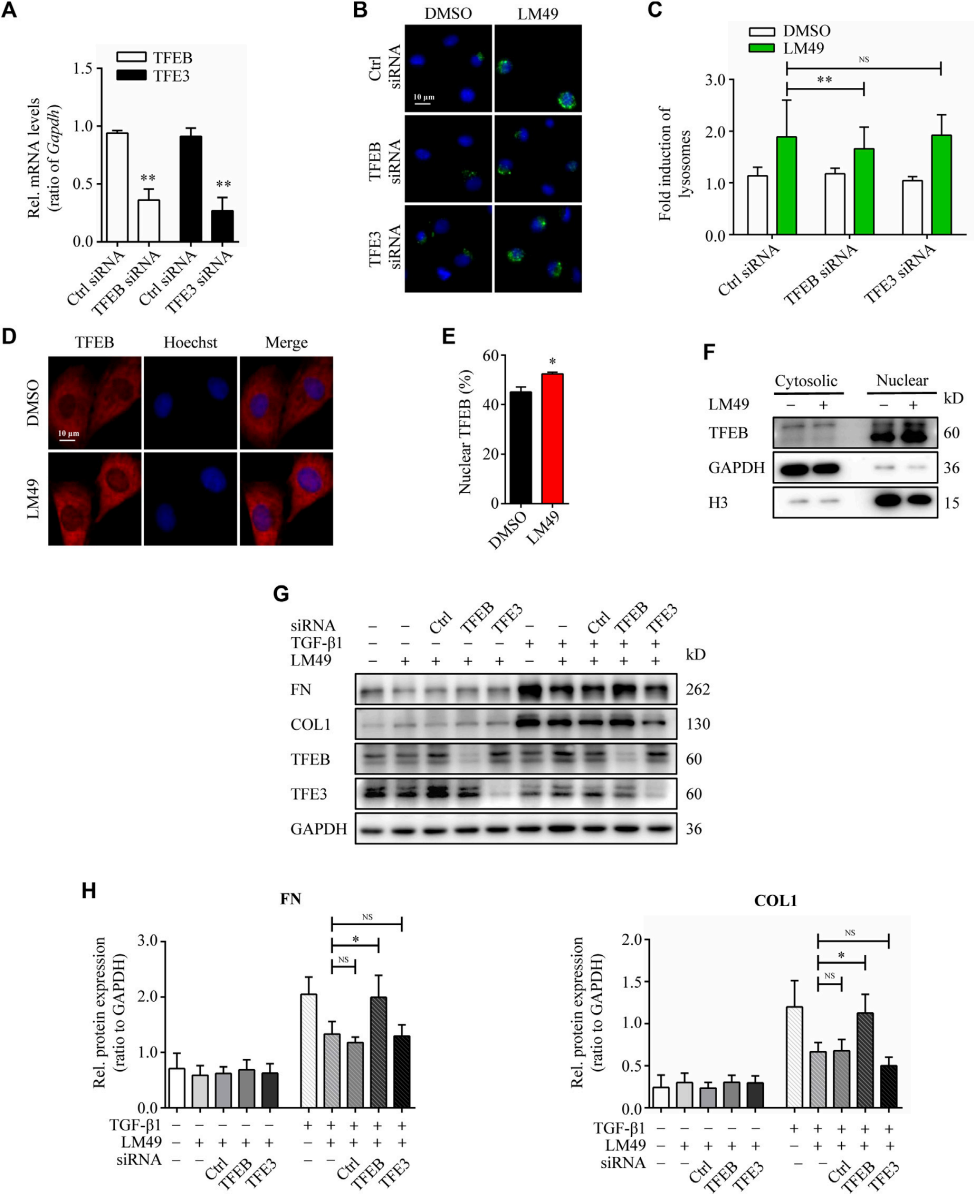
TFEB-dependent lysosome biogenesis is required for LM49-induced ECM protein degradation. **(A)** Quantitative RT-PCR analysis of the mRNA levels of TFEB and TFE3 in NRK49F cells treated with control (Ctrl) siRNA, TFEB siRNA or TFE3 siRNA. **p < 0.01 compared with Ctrl siRNA group (n = 3). **(B)** LysoTracker Green staining in DMSO-treated or LM49 (20 μM)-treated NRK49F cells transfected with Ctrl siRNA, TFEB siRNA or TFE3 siRNA. (Scale bar, 10 μm). **(C)** Quantification of LysoTracker Green intensity of **(B)**. **p < 0.01 compared with Ctrl siRNA + LM49 group (n = 3) **(D)** Immunostaining of TFEB analysing the subcellular localization in NRK49F cells treated with DMSO or LM49 (20 μM) for 24 h (Scale bar, 10 μm). **(E)** Quantification of the ratio of nuclear TFEB to total TFEB in **(D)**. *p < 0.05 compared with DMSO group (n = 3). **(F)** Immunoblot analysis of the protein expression of cytosolic and nuclear TFEB in NRK49F cells treated with DMSO or LM49 (20 μM) for 24 h. **(G)** Immunoblot analysis of the protein expression of FN and COL1 in DMSO-treated or LM49 (20 μM)-treated NRK49F cells transfected with Ctrl siRNA, TFEB siRNA or TFE3 siRNA in the absence or presence of TGF-β1 (5 ng/ml) for 24 h. **(H)** Quantification of FN and COL1 intensity in **(G)**. *p < 0.05 compared with LM49+TGF-β1 group (n = 3).

Lysosome biogenesis can be triggered by the transcription factors TFEB and/or TFE3, which increase the number of lysosomes and promote degradation(Li et al., 2016 ). To investigate the role of TFEB and TFE3 in LM49-induced biogenesis of lysosome, we knocked down TFEB and TFE3 expression by small interfering RNA (siRNA). Compared to control cells, siRNA knockdown of TFEB, but not TFE3, significantly inhibited the LM49-induced lysosome increase (Figures 4A–C). Moreover, LM49 induced nuclear translocation of TFEB in NRK49F cells (Figures 4D–F). Thus, LM49 induces lysosome biogenesis specifically through TFEB. Next, we explored whether TFEB is required for LM49-induced ECM protein degradation. Knockdown of TFEB with siRNA, rather than TFE3, reversed LM49-induced FN and COL1 degradation (Figures 4G,H). Taken together, these results show that LM49 increases lysosome biogenesis and induces ECM degradation via nuclear translocation of TFEB.

### LM49 induces ECM protein degradation by GSK3β

**FIGURE 5:**
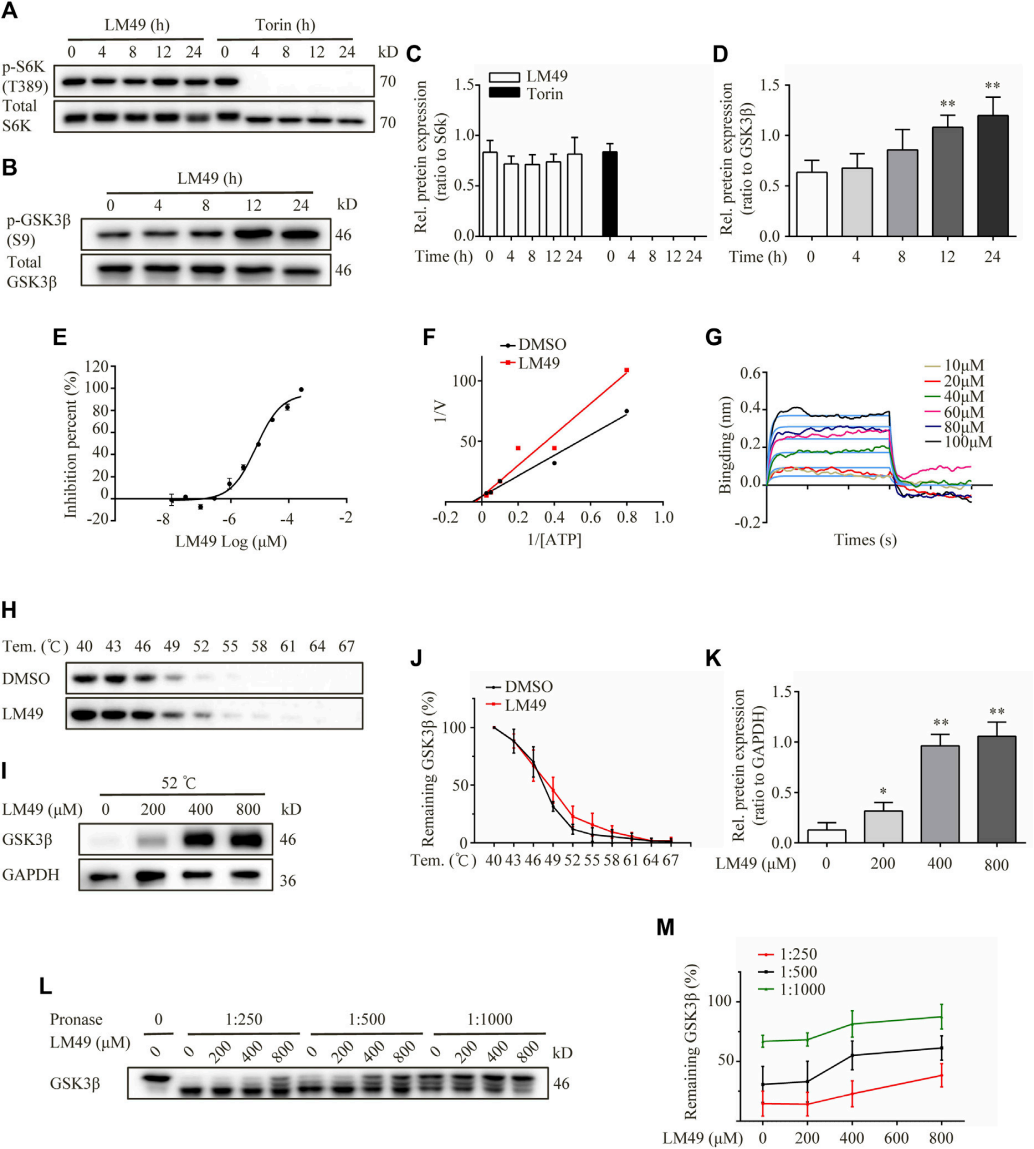
LM49 is a new GSK3β inhibitor. **(A)** Immunoblot analysis of phosphorylation of S6K in NRK49F cells treated with LM49 (20 μM) or Torin1 (1 μM) for the indicated time points. **(B)** Immunoblot analysis of phosphorylation of GSK3β in NRK49F cells treated with LM49 (20 μM) for the indicated time points. **(C)** Quantification of phosphorylation of S6K in **(A)**. (n = 3). **(D)** Quantification of phosphorylation of GSK3β in **(B)**. **p < 0.01 compared with 0 h group (n = 3).**(E)** Kinase activity of GSK3β in different concentrations of LM49. **(F)** Lineweaver-Burk plots. **(G)** BLI analysis to measure dissociation kinetics of LM49 toward GSK3β. **(H)** CETSA confirms the direct binding of LM49 to GSK3β in various temperatures. **(I)** CETSA confirms the direct binding of LM49 to GSK3β in the presence of increasing concentrations of LM49. **(J)** Quantification of GSK3β intensity in **(H)**. Each group was normalised as a percentage of that at 40°C. (n = 3). **(K)** Quantification of GSK3β intensity in **(I)**. *p < 0.05, **p < 0.01 compared with 0 μM group (n = 3). (L) DARTS confirms the direct binding of LM49 to GSK3β. **(M)** Quantification of GSK3β intensity in **(L)**. Each group was normalised as a percentage of that without pronase. (n = 3).

TFEB activity is regulated by mTORC1 (Li et al., 2016). Therefore, we examined whether LM49 inhibits mTOR activity. Whereas Torin1 clearly inhibited the phosphorylation of ribosomal S6 kinase (S6K), a known mTOR substrate, LM49 had no effect on S6K phosphorylation (Figures 5A–C). Thus, we surmised that LM49 induces mTORC1-independent TFEB nuclear translocation.

Phosphorylation of TFEB by GSK3β is important for cytoplasmic sequestration of TFEB, which controls lysosome biogenesis, independently of mTORC1 (Li et al., 2016). To determine whether GSK3β signalling is involved in LM49-induced lysosome biogenesis, we examined GSK3β phosphorylation and kinase activity. We monitored an antibody recognising Ser9-phosphorylation of GSK3β, and found that phosphorylation was increased by LM49 treatment, suggesting that LM49 induced the inactivation of GSK3β (Figures 5B–D). Moreover, the kinase activity of GSK3β was significantly inhibited by LM49 *in vitro*, in comparison with DMSO as negative control and staurosporine as positive control (Table 1). LM49 exhibited a concentration-dependent increase in the inhibition of GSK3β kinase activity and showed a half maximal inhibitory concentration (IC_50_) of 9.28 μM (Figure 5E). To clarify the inhibition mechanism of LM49 on GSK3β, Lineweaver-Burk analysis were performed. LM49 had the same y-intercept as DMSO, but there were different slopes and x-intercepts between the two data sets (Figure 5F), suggesting that LM49 is an ATP-competitive inhibitor of GSK3β.To further confirm the interaction between LM49 and GSK-3β, we performed a BLI assay to measure the dissociation kinetics. BLI analysis revealed that LM49 binds to GSK3β in a concentration-dependent manner, with an equilibrium dissociation constant (K_D_) value of 1.22 × 10^–4^ M (Figure 5G). Drug affinity responsive target stability (DARTS) and Cellular thermal shift assay (CETSA) are label-free approaches to evaluate the binding of small molecules to potential protein targets, based on the assumption that ligand-bound proteins show increased stability and protection against proteolytic and thermal treatment (Chang et al., 2016). We found that GSK3β was almost completely protected from degradation at a 1: 1,000 protease-to-cell lysate ratio in the presence of 800 μM LM49 (Figures 5L,M). As the temperature was increased, the disappearance of GSK3β in the LM49-treated group was slower than in the DMSO-treated group (Figures 5H–J). A decrease of GSK3β degradation was observed at 52°C, as LM49 concentration increased (Figures 5I–K).

**TABLE 1:** GSK3β kinase activity measured by Off-chip Mobility Shift Assay.

Kinase	Sample	Concentration [μmol/L]	Signal [conversion %]	% Inhibition
GSK3β (-)	DMSO		2.3 ± 0.2	
GSK3β (+)	DMSO		54.2 ± 0.9	
GSK3β (+)	Staurosporine	10	1.8 ± 0.7	101.0 ± 1.4
GSK3β (+)	LM49	10	26.7 ± 0.3	52.9 ± 0.6

**FIGURE 6:**
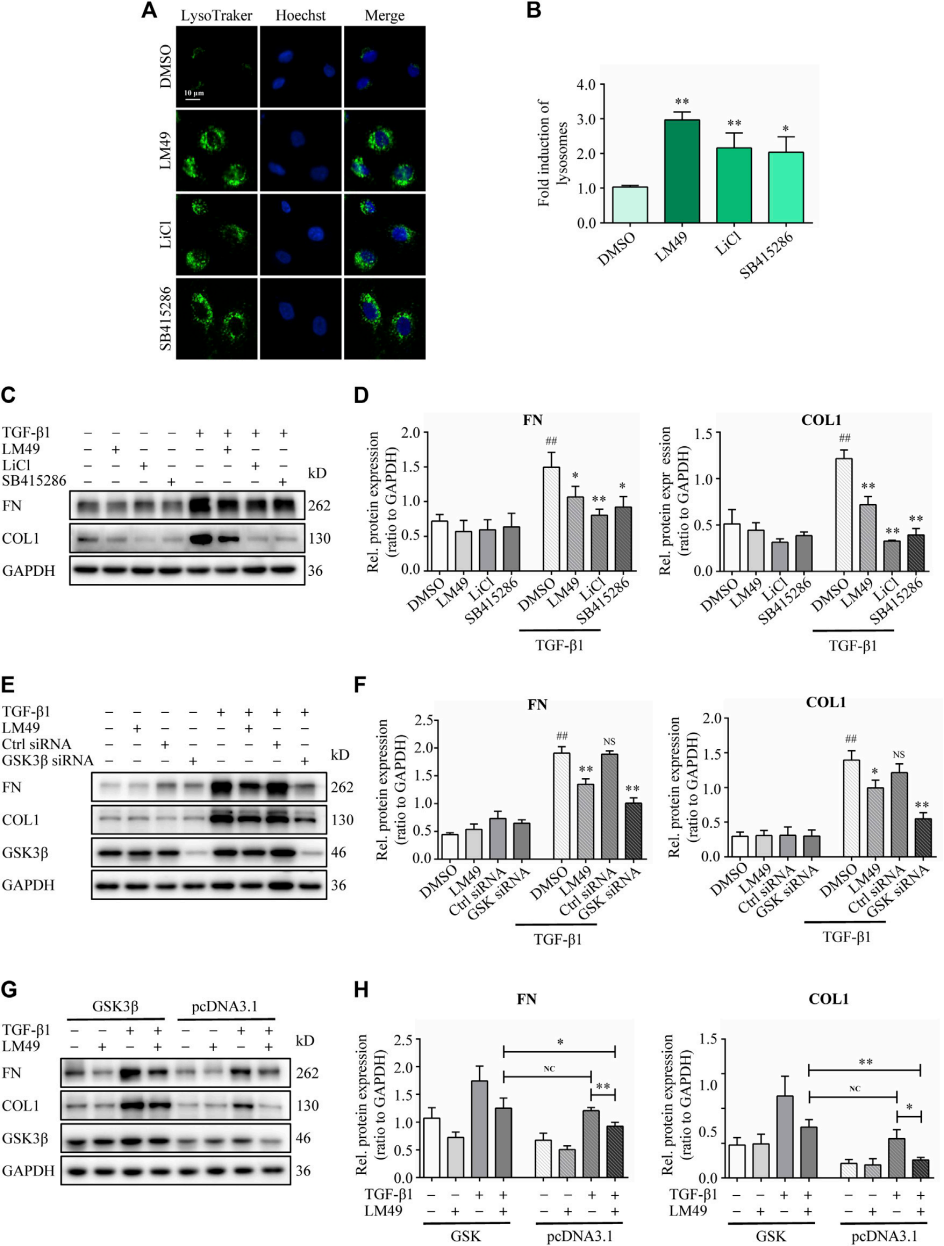
LM49 acts through GSK3β to induce ECM protein degradation. **(A)** LysoTracker Green staining in NRK49F cells treated with LM49 (20 μM, 24 h), LiCl (10 mM 24 h) and SB415286 (20 μM, 24 h). (Scale bar, 10 μm). **(B)** Quantification of LysoTracker Green intensity of **(A)**. *p < 0.05, **p < 0.01 compared with DMSO group (n = 3). **(C)** Immunoblot analysis of the protein expression of FN and COL1 in NRK49F cells treated with LM49 (20 μM), LiCl (10 mM), or SB415286 (20 μM) in the absence or presence of TGF-β1 (5 ng/ml) for 24 h. **(D)** Quantification of FN and COL1 intensity in **(C)**. ^##^ *p* < 0.01 compared with DMSO group, *p < 0.05, **p < 0.01 compared with TGF-β1 group (n = 3). **(E)** Immunoblot analysis of the protein expression of FN and COL1 in DMSO-treated or LM49 (20 μM)-treated NRK49F cells transfected with Ctrl siRNA or GSK3β siRNA in the absence or presence of TGF-β1 (5 ng/ml) for 24 h. **(F)** Quantification of FN and COL1 intensity in **(E)**. ^##^ *p* < 0.01 compared with DMSO group, *p < 0.05, **p < 0.01 compared with TGF-β1 group (n = 3). **(G)** Immunoblot analysis of the protein expression of FN and COL1 in DMSO-treated or LM49 (20 μM)-treated NRK49F cells transfected with pcDNA 3.1 or pcDNA 3.1- GSK3β in the absence or presence of TGF-β1 (5 ng/ml) for 24 h. **(H)** Quantification of FN and COL1 intensity in **(G)**. *p < 0.05, **p < 0.01 compared with TGF-β1+pcDNA3.1 group or LM49+TGF-β1+pcDNA3.1 group (n = 3).

To determine whether LM49 induced ECM protein degradation through direct inhibition of GSK3β kinase activity, the GSK3β inhibitors LiCl and SB415286 were used. Both LiCl and SB415286 increased lysosome biogenesis (Figures 6A,B), and decreased TGF-β1-induced FN and COL1 protein levels in NRK49F cells (Figures 6C,D), consistent with LM49. Moreover, knocking down GSK3β also reduced FN and COL1 protein deposition in the presence of TGF-β1 stimulation (Figures 6E,F). To further confirm the role of GSK3β in LM49-mediated ECM protein reduction, we overexpressioned GSK3β in NRK49F cells. When transfected with pcDNA3.1, LM49 decreased TGF-β1-induced FN and COL1 deposition, however, this reduction was antagonized by the overexpression of GSK3β, which nearly returned to the level of only TGF-β1 treatment (Figures 6G,H). These findings suggest that LM49 acts through GSK3β to induce ECM protein degradation.

### Molecular docking and molecular dynamics simulation analysis of LM49 and GSK3β

**FIGURE 7:**
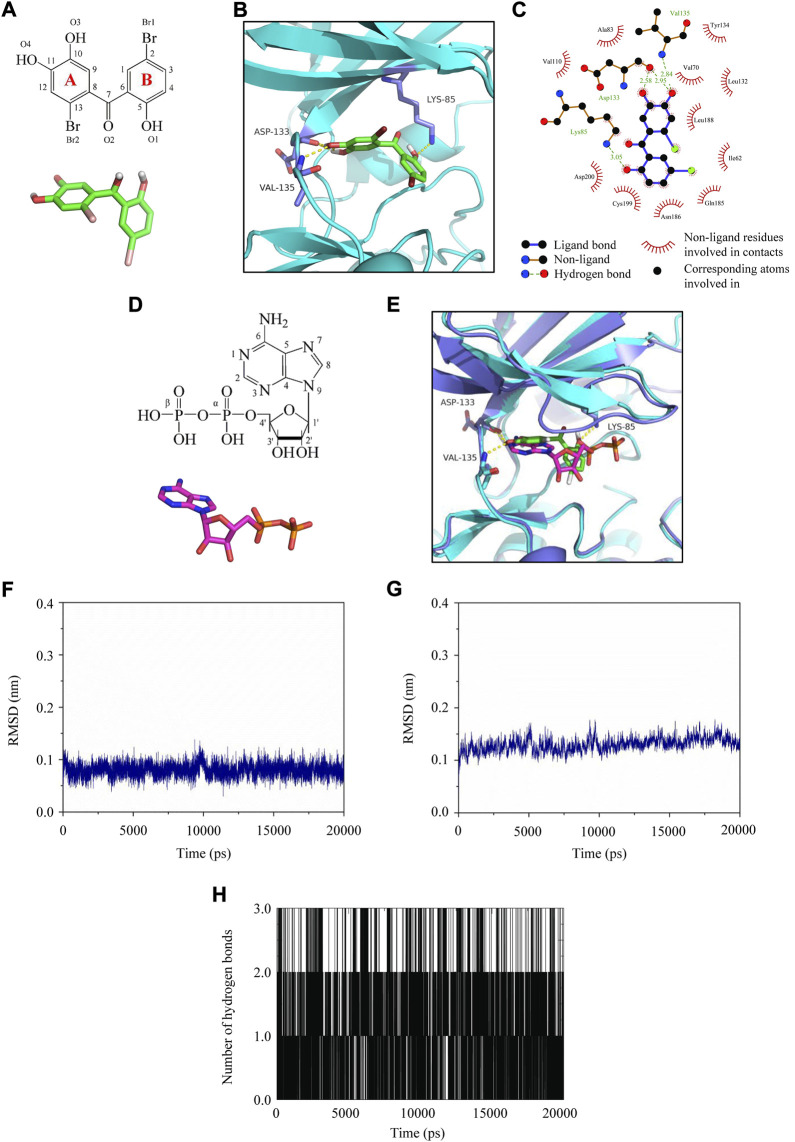
Molecular docking and molecular dynamics simulation analysis of LM49 and GSK3β. **(A)** Chemical structure and interactive chemical structure of LM49. **(B)** Binding mode of LM49 and GSK3β analysed with Autodock4.2. Hydrogen bonds are indicated by the yellow dashed lines. **(C)** Binding mode of LM49 and GSK3β analysed with LigPlot. **(D)** Chemical structure and interactive chemical structure of ADP. **(E)** Superposed GSK3β structure in complex with LM49 (green backbone) and ADP (magentas backbone). Hydrogen bonds are indicated by the yellow dashed lines. **(F)** RMSD of LM49 obtained during the 20 ns MD simulation. **(G)** RMSD of GSK3β backbone obtained during the 20 ns MD simulation. **(H)** Hydrogen bond map of LM49 and GSK3β during the 20 ns molecular dynamics simulation.

To further elucidate the binding mode between LM49 and GSK3β at the molecular level, molecular docking was conducted. LM49 occupied the cleft formed between the N- and C-lobes of GSK3β, which is the hydrophobic pocket for ATP binding (Figures 7A–C). The hydroxyl groups at C-10 and C-11 of the LM49 “A” ring make hydrogen bonds with the Asp133 backbone carbonyl group. The hydroxyl group at C-11 also makes a hydrogen bond with the Val135 backbone amide group (Figures 7B–C). The hydroxyl group at the C-5 of the “B” ring makes a hydrogen bond with the side chain of Lys85 (Figures 7B–C). In addition to the polar interactions, LM49 also makes hydrophobic contacts with GSK3β residues Ile62, Val70, Ala83, Val110, Leu132, Tyr134, Gln185, Asn186, Leu188, Cys199, and Asp200 (Figure 7C).

Prior to crystallisation, ATP was added to the protein preparation and allowed to hydrolyse. During crystallization, a molecule of ADP is sandwiched between the N- and C-terminal lobes in many structures (Stamos et al., 2014). The structural superposition onto the GSK3β structure with ADP (PDB code: 4NM3) and LM49 revealed that the “A” ring of LM49 was structurally comparable to the adenine group of ADP while the “B” ring was analogous to the ribose group and α-phosphate (Figures 7D,E). The hydroxyl groups in the LM49 “A” ring and the amine group of C-6 in adenine were close and interact with the backbone atoms of Asp133 and Val135. The hydroxyl group at C-5 in the LM49 “B” ring overlaps with the oxygen atom in the α-phosphate that interacts with the amine group in the side chain of Lys85 (Figures 7D,E). This result revealed that LM49 inhibits GSK3β by binding to the ATP pocket, which is consistent with the result of the kinase activity experiments.

Molecular dynamics simulation was performed to evaluate the structure stability and the hydrogen bond interaction of LM49 and GSK3β. After fine-tuning, the structure of LM49 (Figure 7F) and GSK3β (Figure 7G) reached a dynamic equilibrium by the end of the simulation. A maximum of three hydrogen bonds were present in the complex of LM49 and GSK3β (Figure 7H). The binding free energy between LM49 and GSK3β was -115.25 kJ/mol (Table 2), as calculated with MM/PBSA methods. Among these, the van der Waals energy and electrostatic energy play a key role for the binding of LM49 and GSK3β, indicating that LM49 could form a stable complex with GSK3β.

**TABLE 2:** Binding free energy of LM49 to GSK3β (kJ/mol).

$∆E_{vdW}$	$∆E_{ele}$	$∆G_{PB}$	$∆G_{SA}$	$∆G_{binding}$
−147.56	−26.16	71.428	−13.15	−11.23

All the energy are in kJ/mol. Snapshots extracted from the last 0.5 ns MD simulation were submitted to MMPBSA.py for the free energy calculation. $∆E_{vdW}$ , van der Waals energy; $∆E_{ele}$ electrostatic energy; $∆G_{PB}$ , polar solvation energy; $∆G_{SA},$ solvent accessible surface area; $∆G_{binding}$ , binding free energy.

## Discussion

This study demonstrated that LM49, a polyphenol compound synthesised by our group, significantly attenuated renal fibrosis by promoting ECM degradation. Renal fibrosis is characterised by an imbalance in ECM homeostasis, and a major goal for antifibrotic therapies is to restore the balance between production and degradation of ECM (Zhao et al., 2019). TGF-β1 signalling is the major driver of renal fibrosis, but direct targeting of TGF-β1 signalling to reduce ECM production has proven difficult due to the involvement of TGF-β1 in many other biological processes in addition to tissue fibrosis (Meng et al., 2016). In this study, LM49 decreased ECM protein expression, but not mRNA expression. The lysosomal inhibitor BafA1 dramatically inhibited LM49-induced proteolysis of COL1 and FN. Furthermore, LM49 triggered the translocation of COL1 to the lysosomes. Thus, LM49 promotes lysosome-dependent degradation of the ECM. ECM degradation is a two-part process: extracellular cleavage by proteolytic enzyme including the metalloproteinases and intracellular uptake by macropinocytic, phagocytic and endocytic pathways for lysosomal degradation(McKleroy et al., 2013; Zhao et al., 2019). Through an increase in the level of lysosomal protein degradation, LM49 reversed the TGFβ1-induced imbalance between ECM production and ECM degradation, which presents a promising strategy for renal fibrosis therapy.

The role of lysosome biogenesis in the regulation of LM49-mediated ECM degradation is an important finding of this work. Increased lysosome biogenesis is considered as a compensatory mechanism to counteract lysosomal dysfunction (Bajaj et al., 2019). The genetic “program” controls lysosome biogenesis and function, providing a potential therapeutic target to enhance cellular clearance and rescue phenotypic abnormalities in lysosomal storage diseases (LSDs), immune diseases, neurodegenerative disorders and cancer (Sardiello et al., 2009; Bonam et al., 2019). Lysosomal dysfunction was also identified in CKD (Hilliard et al., 2009; Medina-Navarro et al., 2019; Kunishige et al., 2020). Changes in albumin’ tertiary structure induced lysosomal dysfunction that associated with interstitial fibrosis and DN progression (Medina-Navarro et al., 2019). In this study, LM49 increased lysosome biogenesis through TFEB. Furthermore, TFEB knockdown with siRNA reversed LM49-induced lysosome biogenesis and ECM degradation. The role of TFEB-mediated autophagy in renal fibrosis has been extensively studied(Tang et al., 2020; Zhang et al., 2020; Yuan et al., 2021), however, there are currently no reports on the role of TFEB-mediated lysosome biogenesis in renal fibrosis. Our findings, that LM49 increases ECM degradation through activating lysosome biogenesis, are of potential interest for kidney fibrosis. Validated targets of treatment have still not been established for renal fibrosis, and enhancement of lysosome biogenesis could be considered as a candidate therapeutic target.

Without inhibiting mTORC1 activity, LM49 promotes TFEB-dependent lysosome biogenesis via GSK3β inhibition. Although mTORC1-mediated phosphorylation at specific serine residues regulates TFEB activity, long-term use of mTORC1 inhibitors results in unpredictable side effects upon the key role of mTORC1 in other cellular biosynthetic pathways (Lamming et al., 2013). Therefore, identification of mTORC1-independent agents that can pharmacologically control TFEB activity offers an alternative avenue to enhance lysosome biogenesis. TFEB phosphorylation by GSK3β promotes the dynamic localization of TFEB on lysosomes, which facilitates mTOR phosphorylation of TFEB, but not affects mTOR activity (Li et al., 2016). GSK3β does not directly participate in the TFEB nuclear localisation may account for the modest effect of LM49 on TFEB. Our finding that TFEB, but not TFE3, regulates the LM49-induced lysosome increase is in accordance with the previous report that GSK3β inhibition specifically activates TFEB rather than TFE3 (Li et al., 2016).

Importantly, we identified LM49 synthesised by our group as a new GSK3β inhibitor. LM49 occupied the hydrophobic pocket of GSK3β that functions in ATP binding and formed stable hydrogen bonds with GSK3β, shown through molecular docking and molecular dynamics simulations. Future work on resolving the co-structure of GSK3β and LM49 will help delineate how LM49 binds to and inhibits GSK3β. Activated GSK3β was elevated in DN mice and type 2 diabetic patients, which is correlated the severity of DN and ECM accumulation (Liang et al., 2020; Abdou and Abd Elkader, 2022). Moreover, the activity of GSK3β was augmented in other CKDs, such as focal segmental glomerular sclerosis (FSGS) (Chen et al., 2021), folic acid (FA) nephropathy (Chen et al., 2021), and chromic allograft nephropathy (Gong et al., 2008; Yan et al., 2012). GSK3β inhibition by lithium has been shown to effectively ameliorate renal fibrosis in FA nephropathy mice (Chen et al., 2021). GSK3β modulates the competition between CREB signalling and TGF-β1/Smad signalling for the recruitment of the shared transcriptional coactivator CBP to drive molecular changes of TEC profibrogenic plasticity and ameliorate renal fibrosis (Chen et al., 2021). Consistent with previous results, this study identified that GSK-3β inhibition reduced TGF-β1-induced ECM deposition. However, in our study, GSK-3β inhibition increased ECM degradation by activating lysosome biogenesis in renal fibroblast cells by a different mechanism. Therefore, LM49 is a potential agent to mitigate renal fibrosis by therapeutically targeting of GSK-3β.

## Conclusion

**FIGURE 8:**
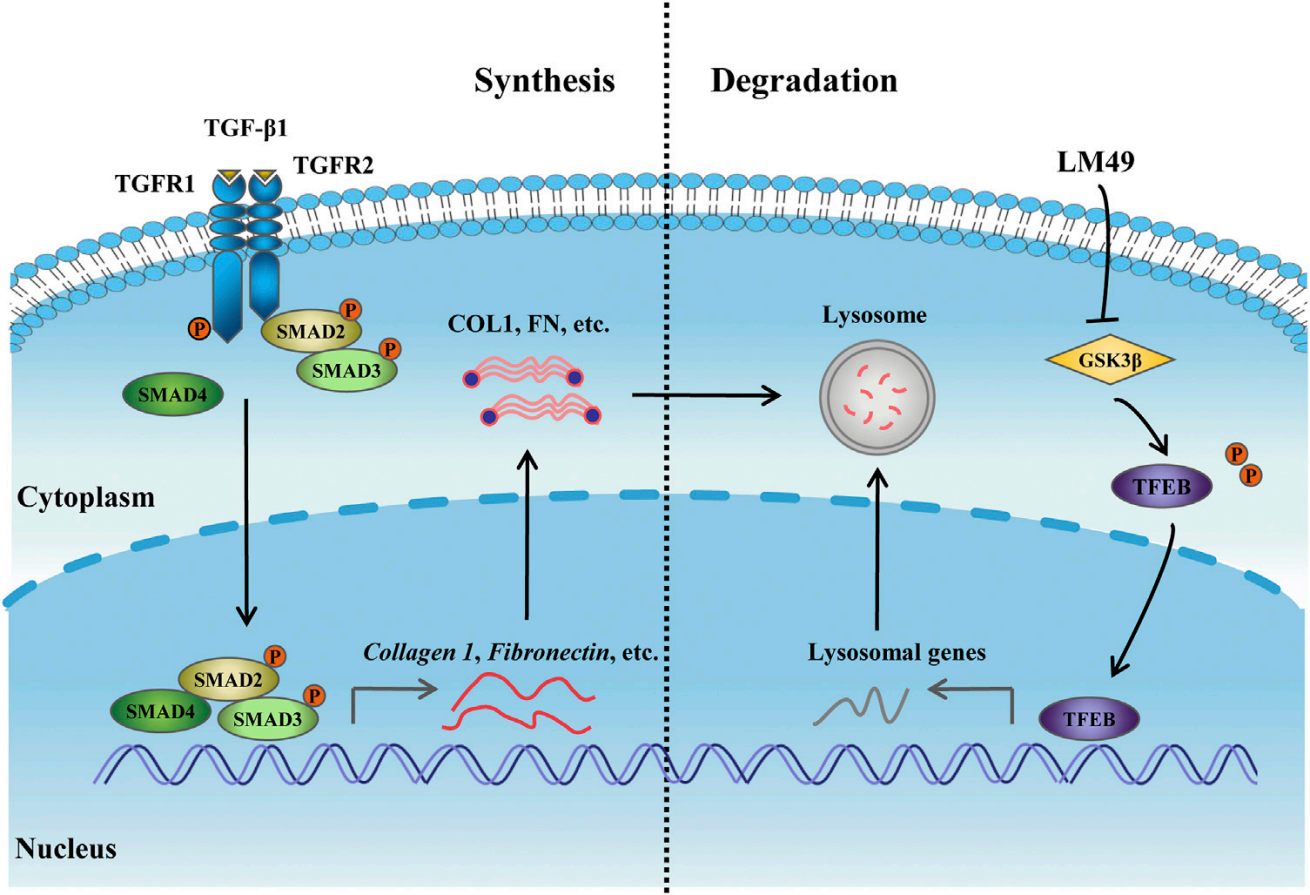
The mechanism of LM49-activated lysosome biogenesis involves directly inhibiting GSK3β to trigger ECM degradation.

Our study shows that LM49 induces lysosome-dependent ECM degradation to restore ECM homeostasis, thereby ameliorating renal fibrosis. Directly inhibiting GSK3β with LM49 triggers TFEB nuclear translocation, which enhances lysosome biogenesis to promote lysosomal degradation of the ECM (Figure 8). These findings provide a novel perspective for treating renal fibrosis by pharmacologically modulating GSK3β-dependent lysosome biogenesis. These results also provide a rationale for the potential application of LM49 as a small-molecule inhibitor that reduces renal fibrosis.

## Data Availability

The original contributions presented in the study are included in the article/Supplementary Material, further inquiries can be directed to the corresponding author.
