# Supplementary material for: GSK3β-dependent lysosome biogenesis: An effective pathway to mitigate renal fibrosis with LM49
Source: Front Pharmacol. 2022 Sep 26;13:925489. doi: 10.3389/fphar.2022.925489 (PMC9550195; doi:10.3389/fphar.2022.925489)
Supplement: Supplementary file 2 [file Table1.DOCX]

Supplementary Table 1 The primers of target genes

| Gene | Primers | |
| --- | --- | --- |
| COL1 | Forward | CGAGTCACACCGGAACTTGG |
|  | Reverse | CCAATGTCCAAGGGAGCCAC |
| FN | Forward | AGGCACAAGGTCCGAGAAGAGG |
|  | Reverse | CATGAGTCATCCGTAGGCTGGTTC |
| ARSA | Forward | CGGTTCGGAATGGGAAGTACAAGG |
|  | Reverse | GACGGTTGGCGGCATGACAG |
| ARSB | Forward | CAACACGGTCCTCATCTTCTCCAC |
|  | Reverse | CTTGCCACGAAACCTGCTCCTC |
| ATP6V0E1 | Forward | GTTGGTGACCTGTTCCGTTTG |
|  | Reverse | GCTTCTAGCTGTGACCTCAGTG |
| ATP6V1H | Forward | TGCCAATGTTGAATCGCCAGGAC |
|  | Reverse | CTGCCTTCCATGAGTTCCTTTCCC |
| CTSA | Forward | GACACAAGGAAGCAGAGATGCC |
|  | Reverse | CAATCGATTTCATCCTGGTCCG |
| CTSB | Forward | AGGCTGGACGCAACTTCTACAATG |
|  | Reverse | CCTCGCTGAACCCAACCCTTTC |
| CTSD | Forward | CCTTCTACCTGAACAGGGACC |
|  | Reverse | CAGTAGGCCTTTCGGGTGAC |
| CTSF | Forward | CACTTGCTGCTGAGGAAGGACTG |
|  | Reverse | ATTGGAAGGAATGCTCGGAAGGTC |
| CLCN7 | Forward | CGGTCACCTCACATTCGCTCAC |
|  | Reverse | CCACGATGATGCCAACCTTCTCTC |
| GALNS | Forward | TCCGCAATGGCTTCTACACAACC |
|  | Reverse | AGGCAGGAGGTGTTCTGAGTTAGG |
| GBA | Forward | TGGGAGCAGAGTGTTCGGTTAGG |
|  | Reverse | GATTCAGGGCAAGGTTCCAGTCAG |
| GLA | Forward | TGATGCCCAGACATTTGCTGACTG |
|  | Reverse | AACCATTCGCCAAGGATACCACAC |
| GNS | Forward | CGCTTATGTGCCCAGTGCTCTC |
|  | Reverse | GGATCTTCTGCCAGGACTTGCTAC |
| HEXA | Forward | AGGTGCCATTGCCGAAAGACTG |
|  | Reverse | TTCCTCTCCTCAGCAGCTCACAG |
| LAMP1 | Forward | TCCTCATCGTCCTCATCGCCTAC |
|  | Reverse | CCTCCCTCCCTTCCACACCTATC |
| MCOLN1 | Forward | GACCCAGCCAACGATACCTTTGAC |
|  | Reverse | CTGCCATCCAAGAAGTCCAAGTCC |
| PSAP1 | Forward | GCCACAGCACAGAGACTTGAGAAG |
|  | Reverse | CGGGAAGACGGGAAAGCAGAATG |
| SCPEP1 | Forward | GACTCAAGCCACGGAACACTACC |
|  | Reverse | TCAGGAGGACCATCATGTCGGAAG |
| TPP | Forward | TCATACCAGGAGGAAGCAGTAGCC |
|  | Reverse | CCACCCAGTAGCCATCAGAAAGC |
